# An Integrated Metabolomic and Genomic Mining Workflow To Uncover the Biosynthetic Potential of Bacteria

**DOI:** 10.1128/mSystems.00028-15

**Published:** 2016-05-03

**Authors:** Maria Maansson, Nikolaj G. Vynne, Andreas Klitgaard, Jane L. Nybo, Jette Melchiorsen, Don D. Nguyen, Laura M. Sanchez, Nadine Ziemert, Pieter C. Dorrestein, Mikael R. Andersen, Lone Gram

**Affiliations:** a Department of Systems Biology, Technical University of Denmark, Kgs. Lyngby, Denmark; b Department of Chemistry and Biochemistry, University of California San Diego, La Jolla, California, USA; c Center for Marine Biotechnology and Biomedicine, Scripps Institution of Oceanography, University of California San Diego, La Jolla, California, USA; d Interfaculty Institute of Microbiology and Infection Medicine, University of Tübingen, Tübingen, Germany; e Collaborative Mass Spectrometry Innovation Center, University of California at San Diego, La Jolla, California, USA; f Skaggs School of Pharmacy and Pharmaceutical Sciences, University of California at San Diego, La Jolla, California, USA; G. W. Hooper Research Foundation

**Keywords:** *Pseudoalteromonas*, comparative genomics, natural products, untargeted metabolomics

## Abstract

Microorganisms are a rich source of bioactives; however, chemical identification is a major bottleneck. Strategies that can prioritize the most prolific microbial strains and novel compounds are of great interest. Here, we present an integrated approach to evaluate the biosynthetic richness in bacteria and mine the associated chemical diversity. Thirteen strains closely related to *Pseudoalteromonas luteoviolacea* isolated from all over the Earth were analyzed using an untargeted metabolomics strategy, and metabolomic profiles were correlated with whole-genome sequences of the strains. We found considerable diversity: only 2% of the chemical features and 7% of the biosynthetic genes were common to all strains, while 30% of all features and 24% of the genes were unique to single strains. The list of chemical features was reduced to 50 discriminating features using a genetic algorithm and support vector machines. Features were dereplicated by tandem mass spectrometry (MS/MS) networking to identify molecular families of the same biosynthetic origin, and the associated pathways were probed using comparative genomics. Most of the discriminating features were related to antibacterial compounds, including the thiomarinols that were reported from *P. luteoviolacea* here for the first time. By comparative genomics, we identified the biosynthetic cluster responsible for the production of the antibiotic indolmycin, which could not be predicted with standard methods. In conclusion, we present an efficient, integrative strategy for elucidating the chemical richness of a given set of bacteria and link the chemistry to biosynthetic genes.

**IMPORTANCE** We here combine chemical analysis and genomics to probe for new bioactive secondary metabolites based on their pattern of distribution within bacterial species. We demonstrate the usefulness of this combined approach in a group of marine Gram-negative bacteria closely related to *Pseudoalteromonas luteoviolacea*, which is a species known to produce a broad spectrum of chemicals. The approach allowed us to identify new antibiotics and their associated biosynthetic pathways. Combining chemical analysis and genetics is an efficient “mining” workflow for identifying diverse pharmaceutical candidates in a broad range of microorganisms and therefore of great use in bioprospecting.

## Abstract (precis)

We here combine chemical analysis and genomics to probe for new bioactive secondary metabolites based on their pattern of distribution within bacterial species. We demonstrate the usefulness of this combined approach in a group of marine Gram-negative bacteria closely related to *Pseudoalteromonas luteoviolacea*, which is a species known to produce a broad spectrum of chemicals. The approach allowed us to identify new antibiotics and their associated biosynthetic pathways. Combining chemical analysis and genetics is an efficient “mining” workflow for identifying diverse pharmaceutical candidates in a broad range of microorganisms and therefore of great use in bioprospecting.

## INTRODUCTION

Microorganisms have remarkable biosynthetic capabilities and can produce secondary metabolites with high structural complexity and important biological activities. Microorganisms in particular have been a rich source of antibiotics (1, 2) and have served as scaffolds for many other types of drugs. Chemical identification of microbial metabolites is a major bottleneck, and tools that can aid in the prioritization of the most prolific microbial strains and attractive compounds are of great interest.

The search for novel chemical diversity can be done “upstream,” at the genome level, or “downstream,” at the metabolite level. Historically, the approach has been to identify target molecules; however, with the availability of genomes at low costs, genome mining has become highly attractive (3–6). Genome mining analyses are greatly aided by several *in silico* prediction tools (7), such as antiSMASH (8, 9) and NaPDoS (10) for secondary metabolite pathway identification. Several studies have explored the general genomic capabilities within a group of related bacteria (11–16), but only a few studies have explored the overall biosynthetic potential and pathway diversity (17–21). Ziemert et al. (18) compared 75 genomes from three closely related *Salinispora* species and predicted 124 distinct biosynthetic pathways, which by far exceeds the 13 currently known compound classes from these bacteria. The study underlined the discovery potential in looking at multiple strains within a limited phylogenetic space, as a third of the predicted pathways were found only in a single strain.

A large potential is found by combining genome mining with the significant advances in analytical methods for compound identification. Building on the versatility, accuracy, and high sensitivity that liquid chromatography-mass spectrometry (LC-MS) platforms have achieved, sophisticated algorithms and software suites have been developed for untargeted metabolomics (22–26). The core of these programs is, first, feature detection (or peak picking), i.e., the identification of all signals caused by true ions (27), and, second, peak alignment, matching identical features across a batch of samples. Today, many programs consider not only the parent mass and the retention time (RT) but also the isotopic pattern, ion adducts, charge states, and potential fragments (27), which greatly improves the confidence in these feature detection algorithms (28). These high-quality data can be combined with multivariate analysis tools, which not only aids analysis and interpretation but also forms a perfect basis for integration with genomic information. Recently, molecular networking has been introduced as a powerful tool in small-molecule genome mining (21, 29, 30). It builds on an algorithm (31, 32) capable of comparing characteristic fragmentation patterns, thus highlighting molecular families with the same structural features and potentially the same biosynthetic origin. This enables the study and comparison of a high number of samples, at the same time aiding dereplication and tentative structural identification or classification (33).

Here, we present an integrated diversity mining approach that links genes, pathways, and chemical features at the very first stage of the discovery process using a combination of publicly available prediction tools and machine learning algorithms. We use genomic data to interrogate the chemical data and vice versa to get an overview of the biosynthetic capabilities of a group of related organisms and identify unique strains and compounds suitable for further chemical characterization. We demonstrate our approach on a unique group of marine bacterial strains all closely related to *Pseudoalteromonas luteoviolacea* based on 16S rRNA gene sequence similarity (34, 35). Previous studies in our lab have shown that it is a highly chemically prolific and diverse species with strains producing a cocktail of the antibiotics violacein and either pentabromopseudilin or indolmycin (36). We use the integrated approach to evaluate the promise of continued sampling and discovery efforts within this species as demonstrated by the finding of an additional group of antibiotics, the thiomarinols.

## RESULTS

**FIG 1:**
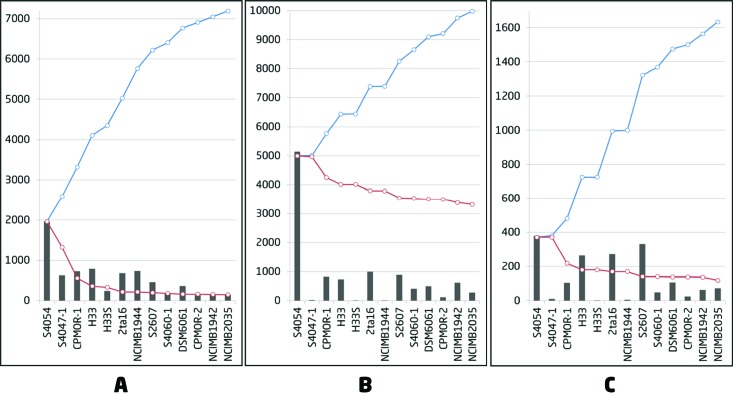
Pan- and core-metabolome and genome plots of 13 *P. luteoviolacea* strains. (A) The pan-metabolome curve (blue) connects the cumulative number of molecular features detected (positive and negative mode merged). The core-metabolome curve (red) connects the conserved number of features. The bars show the number of new molecular features detected in each extract (medium components excluded). (B) Pan-genome (blue) and core-genome (red) curves for all predicted genes. (C) Pan-genome (blue) and core-genome (red) curves for genes predicted to be involved in secondary metabolism.

Thirteen closely related strains previously identified as *P. luteoviolacea* by gene sequence similarity (36) were analyzed for their genomic potential and ability to produce secondary metabolites. The bacteria were cultivated on a complex medium known to support production of secondary metabolites (37) and extracted sequentially by ethyl acetate and butanol to obtain broad metabolite coverage. To obtain a global, unbiased view of the metabolites produced, molecular features were detected by LC-electrospray ionization (ESI)–high-resolution MS (HRMS) in an untargeted metabolomics experiment. On average, more than ~2,000 molecular features were detected in each strain. Merging of ESI^+^/ESI^−^ data resulted in a total of 7,190 features from the 13 strains (excluding medium components), with more features detected in positive mode (6,736) than negative mode (2,151). To facilitate comparison to genomic data, the features were represented as pan- and core plots commonly used for comparative microbial genomics (38, 39). Here, core-metabolome features are shared between all strains, while the pan-metabolome represents the total repertoire of features detected within the collection (Fig. 1A).

Surprisingly, only 2% of the features were shared between all the strains. In contrast, 30% of all features were unique to single strains. As the number and detection of features in each strain change with the chosen threshold for feature filtering, the pan- and core plots were also made based on the 2,000 and 500 most intense features, respectively (see Fig. S1 in the supplemental material). Here, the same trend was observed with 6 to 10% core features and 20% unique features. Thus, regardless of feature filtering settings, the overall pattern of diversity is the same.

10.1128/mSystems.00028-15.2 Figure S1  (A) The pan-metabolome curve (blue) connects the cumulative number of the total number of molecular features detected (positive and negative mode merged). The core-genome curve (red) connects the conserved number of features. The bars show the number of new molecular features detected in each extract (medium components excluded). (B) Pan-metabolome (blue) and core-metabolome (red) curves of the 2,000 most intense features. (C) Pan-metabolome (blue) and core-metabolome (red) curves of the 500 most intense features. Download Figure S1, DOCX file, 0.2 MB. Copyright © 2016 Maansson et al. 2016 Maansson et al. This content is distributed under the terms of the Creative Commons Attribution 4.0 International license.

To link the chemical diversity to the genomic diversity in these closely related strains, we analyzed the 13 genomes by different comparative approaches. The average genome size was approximately 6 Mb with approximately 5,100 putative protein-encoding genes per strain (see Table S1 in the supplemental material). The corresponding pan- and core-genomic analysis was performed using CMG-biotools (39) (Fig. 1B). A total of 9,979 protein-encoding genes were predicted in the pan-genome, including 3,322 genes (33%) conserved between all strains; thus, on average, the core genome constituted ~65% for each strain. Of the accessory genome, 23% of the total genes (2,329) could be found only in a single strain (singletons/unique genes). Considering only genes predicted to be involved in secondary metabolism, the diversity was even higher (Fig. 1C). On average, 8.6% of the total genes were predicted to be allocated to secondary metabolism (see Table S1), which is extremely high compared to other sequenced strains belonging to *Pseudoalteromonas* (40, 41). Similar to the total pan-genome, 24% (386) of the genes putatively involved in secondary metabolism were found in only a single strain; however, only 7% (119) were shared between all 13 strains. Thus, we see approximately a 5-fold-higher genetic diversity in secondary metabolism compared to the full pan-genome.

10.1128/mSystems.00028-15.7 Table S1  Overall descriptive features of all 13 draft genomes. Total genes were predicted using Prodigal 2.00, while antiSMASH 2.0 (8, 9) was used to predict the number of genes allocated to secondary metabolism. *, the total number of OBUs (in parentheses) and number of PKS/NRPS pathways were calculated based on antiSMASH and NaPDoS (10) predictions and recursive analysis by MultiGeneBlast (81). Download Table S1, DOCX file, 0.1 MB. Copyright © 2016 Maansson et al. 2016 Maansson et al. This content is distributed under the terms of the Creative Commons Attribution 4.0 International license.

The high number of unique genes and molecular features suggests an open pan-genome/metabolome (38) in which there is a continuous increase in diversity with continued sampling, which is very attractive for discovery purposes. Both sets of data suggest that 90% of the diversity/genomic potential for secondary metabolism can be covered with 10 strains but that each new strain holds promise for new compounds and biosynthetic pathways.

### Pan-genomic diversity and pathway mapping suggest a highly dynamic accessory genome.

**FIG 2:**
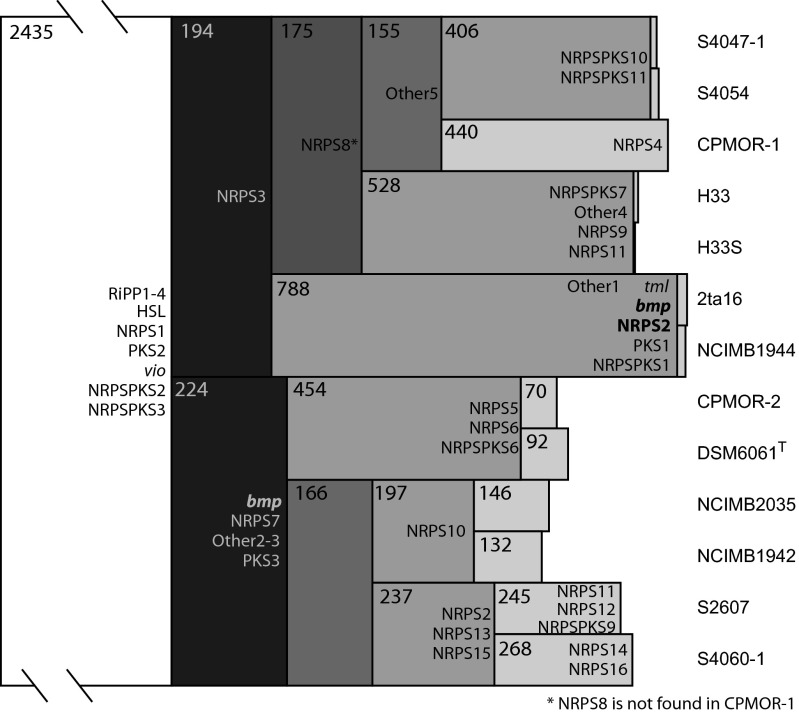
Icicle plot of shared genes for groups of species with OBUs overlaid. The numbers in the boxes show the number of mutual 1:1 orthologs found in the species to the right of that box. The areas of the individual boxes are proportional to the number of genes.

To determine the potential evolutionary relationship between the strains and associated pathways, a pan-genomic map was generated illustrating shared orthologs between groups of species (Fig. 2).

The method uses a conservative BLAST-based nongreedy pairing of genes, which results in 2,435 genes found to be present as 1:1 orthologs in all strains, which is slightly fewer than the 3,388 genes found in the method illustrated in Fig. 1. In general, we observed two main clades based on shared genes, one consisting of six strains and the other of seven. Each clade has 190 to 220 genes unique for that clade. The method also further reflects the genetic diversity of each strain, as illustrated in Fig. 1B and C. Based on the shared orthologs, we generated presence/absence patterns for all genes showing in which other strains that gene has orthologs, a useful starting point for data correlation.

For genetic analysis of biosynthetic pathways in multiple strains, pathways were predicted using antiSMASH across the 13 strains and grouped into 37 operational biosynthetic units (OBUs) (18) (see Table S2 in the supplemental material). OBU presences were compared to the pan-genomic map (Fig. 2) to trace biosynthetic pathways. Only 10 pathways were conserved in all strains, including a glycosylated lantipeptide (RiPP1) and two bacteriocins (RiPP2 and RiPP3). All strains maintained essential pathways likely responsible for production of siderophores (NRPS1 putative catechol-based siderophore) and homoserine lactones (different variations). The violacein pathway *vio* is also conserved in all strains (consistent with the purple phenotype of the pseudoalteromonads), in addition to an unassigned type III polyketide synthase (PKS) and a hybrid nonribosomal peptide synthetase (NRPS)-PKS pathway. Interestingly, the majority of clusters follow the strain lineage suggested by Fig. 2, suggesting that many of the pathways have been introduced and retained based on a competitive advantage of those clusters. More than 50% of the predicted pathways are restricted to one or two strains, suggesting that many pathways are introduced highly dynamically (in evolutionary scale). Whether gene gain or gene loss is responsible for the patchy distribution for most of these OBUs is unclear and was not part of this study. However, evolutionary studies in other organisms have proven that horizontal gene transfer is an important part of the evolution of secondary metabolite clusters (18, 42–45). The exact mechanism of the transfer is not known. No significant amount of transposases or other mobile elements has been found within or in the direct vicinity of the clusters.

10.1128/mSystems.00028-15.8 Table S2  Pathway (OBU) distributions among the 13 *Pseudoalteromonas luteoviolacea* strains and their tentative functionality as predicted by antiSMASH. *, partial pathways on split contigs. Partial pathways with the same pattern of conservation are combined in order to avoid overestimation of diversity. Download Table S2, DOCX file, 0.1 MB. Copyright © 2016 Maansson et al. 2016 Maansson et al. This content is distributed under the terms of the Creative Commons Attribution 4.0 International license.

### Key discriminative metabolites are revealed through feature prioritization and dereplication of the pan-metabolome by SVM and molecular networking.

To explore the diversity within the pan-metabolome and prioritize chemical features for more detailed structural analysis, a two-pronged approach was used: multivariate analysis based on machine learning algorithms and comparative analyses based on the pattern of conservation generated from the pan-genomic diversity map. A classifier based on a combination of a genetic algorithm (GA) and support vector machine (SVM) (46, 47) was used as a feature selection method to filter the most important features from the complex data set, starting with the 500 most intense features and reducing it to the 50 most significant features to distinguish all 13 strains (see Table S3 in the supplemental material). In addition, extracts from all strains were analyzed with LC-ESI-MS/MS to generate a molecular network (see Fig. S2a for full details) (30). The candidates identified by multivariate and comparative analyses were correlated with the molecular network (29, 33) for dereplication and connection of molecular features that likely belong to the same structural class and thus biosynthetic pathway. For example, the *vio* pathway (48) was found in all 13 strains, and the antibiotic violacein was a discriminating core feature (see Table S3). In the molecular network, violacein was found to belong to a molecular family of a minimum of five related analogues (see Fig. S2b) likely associated with the *vio* pathway, including proviolacein and oxyviolacein, as well as a novel analogue with two extra hydroxyl groups.

10.1128/mSystems.00028-15.3 Figure S2  (a) Molecular network of 13 strains of *P. luteoviolacea* based on LC-ESI^+^–MS/MS. Spectra originating from blank medium samples are excluded from the analysis. Highlighted are the three gene cluster family-molecular family pairs identified in this study; those are violacein, indolmycin, and thiomarinol. (b) (A) Molecular network of the violacein molecular family. Gray nodes are shared between all strains, while white nodes are shared among multiple, but not all, strains. Dashed nodes indicate a novel analogue. (B) Selected zoom of MS/MS spectra of violacein (top) with parent mass [M + H]^+^ of 344 Da and the novel analogue (bottom) with an extra hydroxyl group [M + H]^+^ of 376 Da. Download Figure S2, DOCX file, 0.4 MB. Copyright © 2016 Maansson et al. 2016 Maansson et al. This content is distributed under the terms of the Creative Commons Attribution 4.0 International license.

10.1128/mSystems.00028-15.9 Table S3  The 50 discriminating molecular features identified with GA/SVM from the 500 most intense features. Molecular formulas are determined with the MassHunter function “Generate formulas,” also considering the isotope pattern of the peak. All tentative identities are based on hits in AntiMarin or Metlin, and the candidates are evaluated based on accurate mass, isotope pattern (in particular for the halogenated compounds), relative retention time, and fragmentation pattern (for Metlin hits). Potential noise refers to unique masses that are detected by the software at high ion counts, yet it is not possible to confirm the identity of the molecular ion (and thus corresponding formulae and mass accuracy) by the presence of adducts or dimers. Download Table S3, DOCX file, 0.1 MB. Copyright © 2016 Maansson et al. 2016 Maansson et al. This content is distributed under the terms of the Creative Commons Attribution 4.0 International license.

### Some strains have lost the ability to produce polyhalogenated compounds.

The discriminating features do not necessarily reflect the same groupings as the genomic analyses. Therefore, they can be used as a tag for identifying the corresponding biosynthetic pathway through correlation with genomic presence/absence patterns. On the list of descriptive features generated using the SVM (see Table S3 in the supplemental material), there are six highly halogenated features that all seem to be restricted to seven strains: CPMOR-2/DSM6061^T^, S2607/S4060-1, NCIMB1944/2ta16, and CPMOR-1. To investigate whether halogenation in general is unique to those strains, a list of features with a high mass defect was made, resulting in more than 40 halogenated compounds (see Table S4) restricted to the seven strains. Most of them had no match to known compounds, but many match the structural scaffolds of polyhalogenated phenols and pyrroles or hybrids thereof (49) and have expected antibacterial activity (50).

10.1128/mSystems.00028-15.10 Table S4  List of halogenated molecular features identified by mass defect screening in MassHunter. The expected mass defect (0.0937 Da with −0.02 Da per 100 Da ± 0.0100 Da) was determined from known halogenated compounds from *Pseudoalteromonas* in AntiMarin. The isotope pattern was used to confirm the presence of halogenations and used to calculate the molecular formula. Tentative identities are based on hits in AntiMarin and evaluated based on accurate mass and isotope pattern. Compounds marked * have no hit but belong to a known class of isomeric compounds. **, peaks have a poor isotope match resulting in ambiguous determination of the formula. Download Table S4, DOCX file, 0.1 MB. Copyright © 2016 Maansson et al. 2016 Maansson et al. This content is distributed under the terms of the Creative Commons Attribution 4.0 International license.

No pathway predicted by antiSMASH had a halogenase incorporated; thus, the pattern of presence in these seven strains was used to probe for associated clusters. Indeed, we found an intact group of 11 genes (including two brominases) conserved in the seven abovementioned strains (see Fig. S3a in the supplemental material). The recently characterized *bmp* pathway corresponds to these genes (*bmp1* to *bmp10*) (49) and is responsible for the production of polybrominated phenols/pyrroles in strain 2ta16 and a putative multidrug transporter (tentatively named *bmp11*). Surprisingly, all 11 genes were also found in NCIMB1942/NCIMB2035, where no halogenated compounds were detected. Incidentally, in both genomes, the cluster is divided across two contigs with the break point being in *bmp1* in both genomes. Should this be an actual physical division of the contig, or an inserted unsequenceable repeat sequence, it could provide an explanation for the lack of halogenated compounds. However, sequencing of the *bmp1* gene in NCIMB2035 revealed a 1-kb insert in the thioesterase (TE) domain of the gene, likely explaining the lack of compounds (J. Busch, V. Agarwal, A. A. El Gamal, B. S. Moore, G. W. Rouse, L. Gram, and P. R. Jensen, unpublished data). Also, *bmp1*, *bmp2*, a part of *bmp7*, and *bmp8* to *bmp11* were found in S4047-1/S4054, which suggests that a common ancestor had an intact *bmp* pathway.

10.1128/mSystems.00028-15.4 Figure S3  (a) Synteny maps of the *bmp* cluster in the genomes of 11 isolates. In two isolates, H33 and H33S, no trace of the pathway was found. Note that in NCIMB1942 and NCIMB2035, the cluster was found distributed across two contigs, in both cases with the gap being in the *bmp1* gene. While this could be a sequencing artifact, no halogenated compounds were found in these two isolates. For S4047-1 and S4054, *bmp3* to *bmp6* and a part of *bmp7* were missing, as well as point mutations (or sequencing errors) in *bmp10*. In all strains, a predicted multidrug transporter was found downstream of *bmp10*, suggesting a possible resistance mechanism (tentatively named *bmp-11*). (b) Synteny plot of *unk* gene cluster from S0454 and indolmycin biosynthetic gene cluster from *Streptomyces griseus* subsp. *griseus* strain ATCC 12648. The figure was constructed using Easyfig 2.2.2 and the tblastx option (83). The BLAST options were set to minimum length of 25, minimum identity of 25%, and maximum E value of 0.001. Download Figure S3, DOCX file, 0.1 MB. Copyright © 2016 Maansson et al. 2016 Maansson et al. This content is distributed under the terms of the Creative Commons Attribution 4.0 International license.

Two of the discriminative features found in the seven strains are two isomeric dimeric bromophenol-bromopyrrole hybrids with eight bromines in total (see Fig. S4 in the supplemental material). The monomers corresponding to the likewise novel “tetrabromopseudilin” are also found in the extract, suggesting that these “*bis*-tetrabromopseudilins” are true compounds rather than artifacts arising from MS insource chemistry. Full structural characterization of these low-proton-density compounds lies beyond the scope of this study but underlines the versatility of the *bmp* pathway and associated chemical diversity.

10.1128/mSystems.00028-15.5 Figure S4  Isotope patterns of C_10_H_5_Br_4_NO (RTs of 14.42, 14.63, and 14.99 min) (A) and C_20_H_8_Br_8_N_2_O_2_ (RTs of 18.39 and 18.66 min) (B) detected in ESI^−^ (top) and the corresponding extracted ion chromatogram (EIC) (bottom) and putative structure of a “*bis*-tetrabromopseudilin.” Download Figure S4, DOCX file, 0.1 MB. Copyright © 2016 Maansson et al. 2016 Maansson et al. This content is distributed under the terms of the Creative Commons Attribution 4.0 International license.

### Identification of the indolmycin cluster shows resistance genes and potential quorum sensing (QS) control.

Strains S4047-1, S4054, and CPMOR-1 are all producing the antibiotic indolmycin, as previously reported (36). Indolmycin was identified by GA/SVM as a discriminating feature for those three strains. In addition to indolmycin, the molecular family consisted of the N/C-demethyl- and N/C-didemethyl indolmycin analogues as well as indolmyceinic acid, a methylated analogue, and two hydroxylated analogues. Most of these analogues have not been reported from microbial sources, and their tentative structures were verified by their MS/MS fragmentation pattern (see Fig. S5 in the supplemental material).

10.1128/mSystems.00028-15.6 Figure S5  (A) Molecular network of the indolmycin molecular family. Dashed nodes indicate a novel analogue. (B) Tentatively identified indolmycin analogues in strains S4047-1, S4054, and CPMOR-1. (C) MS/MS spectra of selected analogues with assigned fragments Download Figure S5, DOCX file, 0.4 MB. Copyright © 2016 Maansson et al. 2016 Maansson et al. This content is distributed under the terms of the Creative Commons Attribution 4.0 International license.

**FIG 3:**
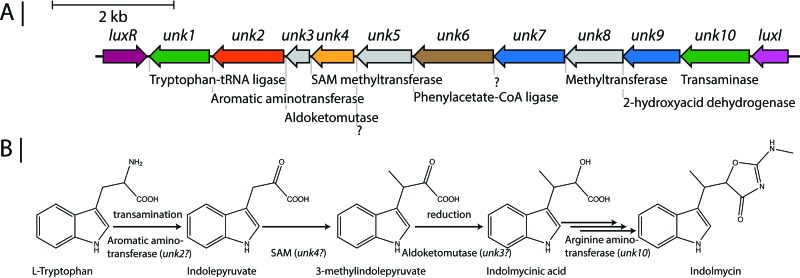
Putative biosynthetic cluster (A) and proposed biosynthetic scheme (B) (51) for indolmycin. CoA, coenzyme A.

Like violacein, indolmycin is derived from l-tryptophan, but even though the biosynthetic pathway has been described by feeding studies in *Streptomyces* (51–53) and recently characterized genetically (54), the biosynthetic cluster responsible has never been characterized. The pan-genome was probed for genes with presence/absence patterns matching the distribution of indolmycin and the related analogues, which led to the identification of 13 clustered genes, suggesting these to be the genetic basis for indolmycin biosynthesis (Fig. 3). The identified genes had predicted functions similar to those expected to be required for the synthesis of indolmycin such as an aromatic aminotransferase (*unk2*), aldoketomutase (*unk3*), *S*-adenosylmethionine (SAM) methyltransferase (*unk5*), and aminotransferase (*unk11*). We have compared our proposed indolmycin biosynthetic gene cluster to that characterized by Du et al. (54) and have identified homologues to the *Streptomyces griseus* ATCC 12648 genes involved in biosynthesis of indolmycin (see Fig. S3b in the supplemental material). Indolmycin has been identified as a competitive inhibitor of bacterial tryptophan-tRNA ligases (55, 56), and the putative cluster seems to incorporate a tryptophan-tRNA ligase (*unk1*), which in *Streptomyces griseus* has been found to confer resistance to indolmycin (56). Interestingly, the cluster in *Pseudoalteromonas* is flanked by *luxI* and *luxR* homologues, something which is not observed in *S. griseus*, suggesting that the indolmycin pathway potentially could be under regulation by quorum sensing.

### Thiomarinols add to the antibiotic cocktail.

The strains 2ta16 and NCIMB1944 were identified as hot spots for biosynthetic diversity based on Fig. 2. This was supported by 313 chemical features (RT and *m/z* pairs) unique to these two strains. Based on the GA/SVM, they can be distinguished from the rest of the strains based on a feature with *m/z* 640 and an RT of 9.73 min (C_30_H_44_N_2_O_9_S_2_), tentatively identified as thiomarinol A. Thiomarinols are hybrid NRPS-PKS compounds based on pseudomonic acid and pyrrothine. One of the gene clusters (hybrid NRPSPKS5) restricted to the pair 2ta16-NCIMB1944 was found to have high similarity to that of pseudomonic acid (*mup*) (57) and the recently characterized thiomarinol (*tml*) cluster (58), corroborating the finding of the compound class. Thiomarinols have previously reported antibacterial activities from *Pseudoalteromonas* sp. strain SANK 73390 (59, 60).

**FIG 4:**
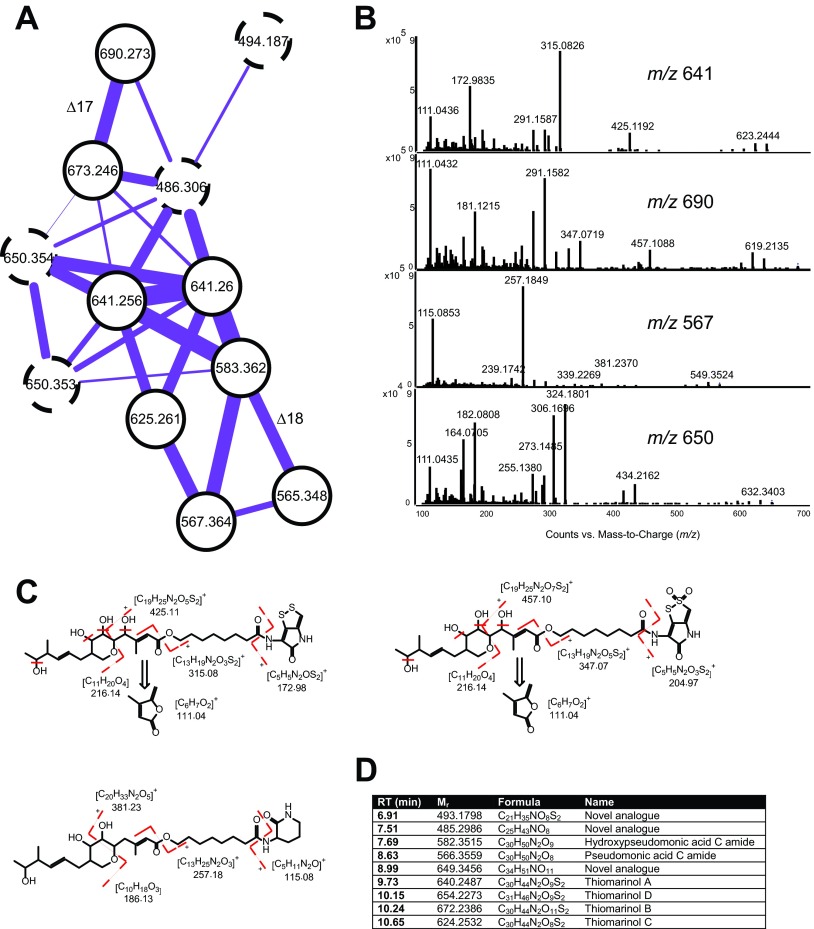
(A) Molecular network of the thiomarinol/pseudomonic acid molecular family. Dashed nodes indicate novel analogues. Mass differences are highlighted for ion adducts only. (B) MS/MS spectra representing the four different analogue types. Parent mass *m/z* 641 is thiomarinol A representing the holothin head type; *m/z* 690 is [M + NH_4_]^+^ of *m/z* 673, thiomarinol B, representing the sulfone head type; *m/z* 567 is pseudomonic acid C amide, representing the nonsulfonated analogues; *m/z* 650 is a novel analogue with a nonsulfonated head. (C) Structures and suggested fragmentation of thiomarinols A and B and pseudomonic acid C amide. (D) Table of detected analogues in strains NCIMB1944 and 2ta16.

In the molecular network, it was possible to identify a whole series of thiomarinol and pseudomonic acid analogues (Fig. 4A and D), all restricted to NCIMB1944 and 2ta16. In addition to thiomarinols A to D, pseudomonic acid C amide and its hydroxyl analogue could be assigned based on the characteristic MS/MS fragmentation pattern (Fig. 4B and C). Besides the known analogues, two novel analogues with formulas C_25_H_43_NO_8_ and C_34_H_51_NO_11_ could be identified. Both shared the marinolic acid moiety based on the C_6_H_6_O_2_ (*m/z* 110.0368) fragment and the loss of C_11_H_2_OO_4_ (*m/z* 216.1362); however, they contained only a single nitrogen and no sulfur, indicating a completely new type of thiomarinol based on neither a holothine nor an ornithine “head” like the known analogues (Fig. 4C).

## DISCUSSION

Advances in genomics and metabolomics have significantly increased our ability to generate high-quality data on microbial secondary metabolism at a very high speed. This, in turn, has enabled a completely new approach to drug discovery combining the two “-omics” approaches.

Using a combination of comparative metabolomics and genomics, we find a high potential and remarkable diversity in terms of secondary metabolite production for strains closely related to *P. luteoviolacea*. Overall, 8.6% of the genes are allocated to secondary metabolism, and on average, 10 NRPS/PKS-related OBUs are predicted. This is very high considering the relatively small size of the genomes (~6 Mb) and is comparable to that of recognized prolific species such as *Salinispora arenicola* (10.9% of 5.8 Mb) (13, 18, 61) and *Streptomyces coelicolor* (8% of 8.7 Mb) (62). Our data suggest an open pan-genome which is characteristic for species that are adapted to several types of environments (63), i.e., being both planktonic and associated with marine macroalgal surfaces. The pan-genome is a dynamic descriptor that will change with the number of strains and the specific subset. Nonetheless, our findings correlate with comparative genomic studies of other bacterial species (11, 12, 14, 63).

We found ~5-fold-higher genetic diversity in secondary metabolism compared to the full pan-genome, which supports the idea that production of secondary metabolites is a functionally adaptive trait (64, 65). More than half of the 41 predicted pathways are restricted to one or two strains, while only 10 pathways were shared between all. This is similar to findings in *Salinispora* (18), where 78% of the pan-genome is associated with one or two strains. Violacein (66, 67), indolmycin (68, 69), and pentabromopseudilin (49) are all examples of cosmopolitan antibiotics found in unrelated species; thus, we hypothesize that *P. luteoviolacea* acquired and retained biosynthetic genes linked to, e.g., antibiotic production as part of adapting to a specific niche that it commonly occupies.

Diversity is further supported at the chemical level. Using unbiased global metabolite profiling, we identify >7,000 putative chemical features among the 13 analyzed strains. As the number of chemical features depends on the filtering threshold, this should not be seen as an absolute number of compounds that can be isolated and fully characterized. However, it provides an unbiased estimate of diversity, which in this case does not seem to change with the chosen threshold. Surprisingly, only 2% of the features were shared between all the strains. To the best of our knowledge, there is only one other similar study on chemical diversity in limited taxonomical spaces approaching the species level. Krug et al. (19, 70) analyzed 98 isolates of *Myxococcus xanthus* in a semitargeted approach and found 11 out of 51 identified compounds to be shared between all strains and a similar fraction present in only one or two strains. We found that almost half of all features and one-third of the 500 most intense features could be assigned to one or two strains (thus taking into account the almost clonal strains), which underlines a great potential for unique chemistry within a group of closely related strains. The detected chemical diversity is higher than what was found on the genetic level, which is to be expected, as the method at this initial screening level does not allow for detecting differential regulation of complete pathways or individual analogues.

The remarkable chemical diversity can be found even within the same sample. Strains S4047, S4054, and S4060 were all collected from seaweed from the same geographical location (37). Strains S4047 and S4054 share 99% of their gene families (clonal) and 70% of their chemical features, but strain S4060 shares only 24% of gene families and 30% of features with the other two. It is also reflected in the biosynthetic pathways, where nine pathways were found in S4060 but not in S4047 and S4054. This is a fascinating ecological conundrum as the accessory metabolites and genes usually are considered to answer the immediate, more localized needs for the strains. Nonetheless, this is not the first report of such an occurrence. Vos and Velicer (71) found 21 genotypes of *M. xanthus* using multilocus sequence typing among 78 strains collected from soil on a centimeter scale. Likewise, significant differences have been found in the chemical profiles of cooccurring strains of *M. xanthus* (19) and *Salinibacter ruber* (72). In contrast, NCIMB1944 and 2ta16, which originate from the Mediterranean Sea (France) and the Florida Keys (United States), respectively, share 99% of their gene families and 70% of their features. That demonstrates that genomic content can be relatively conserved across biogeographical locations, suggesting a high selective pressure to conserve those genes despite an overall low degree of chemoconsistency.

In this study, SVM was applied in conjunction with GA to compile a list of 50 chemical features of interest for further structural characterization. Based on SVM, the reduced set of features are the ones that maximize the difference between samples, which in this study is exploited to select features unique to each strain or a subset of strains. GA works as a wrapper to select features to be evaluated in the SVM classifier (73). The intrinsic nature of the GA makes it highly suitable for discovery purposes as it favors diversity in how the subset of features is selected (47). To the best of our knowledge, there are only a few examples of the use of SVM in untargeted secondary metabolite profiling (74, 75). The list of discriminating features highlights key metabolites, both in the core and in the accessory metabolome. Of the 50 discriminating features, only 15 could be tentatively assigned to known compound classes. In this specific case, the list even reflects the four antibiotic classes identified in this species, underlining the utility of GA/SVM to prioritize not only strains but also compounds before the rate-limiting step of structural identification. The combination with molecular networking further strengthens this approach as it makes it possible to identify structural analogues that likely have similar biological activities.

This is the one of the first examples (20, 21, 29) of direct coupling of genomic and metabolomic data at a global level and at this early stage of the discovery process. By solely using the patterns of presence/absence across the pan-genome in conjunction with synteny, we could identify gene clusters without relying on the functions. This allowed for the identification of the pentabromopseudilin and indolmycin gene clusters. Combined with presence/absence of molecular features, this is an extremely powerful tool for translation back and forth between the genome and metabolome. Thus, it is possible to identify specific compounds using genomic queries or to specifically identify a gene cluster based on chemistry. Of course, in order to fully confirm the link between a compound and its genes, knockout mutants need to be analyzed or entire pathways recombinantly expressed, but here, single candidates for clusters could be directly and rapidly identified.

The combination of metabolomics and genomic data identifies obvious hot spots for chemical diversity among the 13 strains, which permits intelligent strain selection for more detailed chemical analyses. By randomly picking a single strain, in the worst case, only 38% of the 500 most intense chemical features (and thus most relevant from a drug discovery perspective) are covered (NCIMB2035). However, when maximizing strain orthogonality by selecting the two strains (NCIMB1944 and CPMOR-1) with the highest number of unique genes, pathways, and chemical features, 82% of the diversity can be covered. This is extremely important as the isolation and full structural characterization of these compounds still represent the greatest bottleneck in the discovery process. This study shows that investigation of multiple closely related strains is a valuable strategy for detection of new compounds and is imperative for uncovering the full biosynthetic potential of a species.

## MATERIALS AND METHODS

### Strains, cultivation, and sample preparation for chemical analyses.

The 13 strains included in the study were collected or donated to us as previously described (36, 37). We did attempt to build a larger collection; however, *P. luteoviolacea* autolyzes very easily, and in most laboratories, it has not been possible to store and revive strains. The strains were cultured in biological duplicates in marine broth (MB; Difco catalog no. 2216) at 25°C (200 rpm) for 48 h before extraction. See details in Text S1 in the supplemental material.

10.1128/mSystems.00028-15.1 Text S1  Supplemental materials and methods. Download Text S1, DOCX file, 0.1 MB. Copyright © 2016 Maansson et al. 2016 Maansson et al. This content is distributed under the terms of the Creative Commons Attribution 4.0 International license.

### LC-MS and LC-MS/MS data acquisition.

LC-MS and MS/MS analyses were performed on an Agilent 6550 iFunnel quadrupole-time of flight (Q-TOF) LC-MS (Agilent Technologies, Santa Clara, CA) coupled to an Agilent 1290 Infinity ultrahigh-performance liquid chromatography (UHPLC) system. Separation was performed using a Poroshell 120 phenyl-hexyl column (Agilent; 250 mm by 2.1 mm; 2.7 µm) with a water-acetonitrile (ACN) gradient. MS data were recorded in both positive and negative electrospray (ESI) mode in the *m/z* 100- to 1,700-Da mass range. Data for molecular networking were collected using a data-dependent LC-MS/MS as reported previously (76) with optimized collision energies and scan speed. See Text S1 in the supplemental material for the full experimental setup, procedures, and method parameters.

### Feature extraction and multivariate analysis.

Extraction of chemical features was performed using MassHunter (Agilent Technologies; v.B06.00) and the Molecular Features Extraction (MFE) algorithm and recursive analysis workflow. Feature lists were imported to Genespring-Mass Profiler Professional (MPP) (Agilent Technologies; v.12.6) and filtered with features resulting from the medium removed. The feature lists from ESI^+^ and ESI^−^ data were merged in a table as generic data and reimported into MPP. The data were then normalized and aligned, resulting in a single list of chemical features for each sample. The list of discriminating features was generated in MPP using a genetic algorithm with a population size of 25, 10 generations, and a mutation rate of 1. The GA was evaluated using the SVM with a linear kernel type with an imposed cost of 100 and a ratio of 1. The feature list was validated via the leave-one-out method. Further details and settings can be found in the supplemental material. All 50 discriminating features (see Table S3 in the supplemental material) were manually verified to be present in the original data sets. Molecular formulas were predicted from the accurate mass of the molecular ion or related adducts (77) as well as the isotope pattern and matched against AntiMarin (v.08.13) and Metlin (78) databases to tentatively assign known compounds.

### Molecular networking.

For molecular networking, raw LC-MS/MS data were converted to .mgf using MSConvert from the ProteoWizard project (79) and analyzed with the algorithm described in the work of Watrous et al. (30). A new, public interface at http://gnps.ucsd.edu has been made public at the time of writing, and the data have been deposited (MSV000078988) in the corresponding database, http://massive.ucsd.edu. Likewise, the annotated MS/MS spectra for all the identified compounds have been uploaded and added to the GNPS spectral library. The network corresponding to a cosine value of more than 0.7 was visualized using Cytoscape 2.8.3 (80).

### DNA extraction, genome sequencing, and assembly.

Cultures were grown in MB for 1 to 2 days, and genomic DNA was isolated using either the JGI phenol-chloroform extraction protocol or the Qiagen 100/G kit. Library preparation and 150-base-paired end sequencing were done at the Beijing Genomics Institute (BGI) on the Illumina HiSeq 2000 system. At least 100-fold coverage was achieved for all genome sequences generated in this study. Raw sequence data for strain 2ta16 were downloaded from http://www.jcvi.org and assembled as described here. Genomes were assembled using CLC Genomics Workbench (v.2.1 for NCIMB2035, 2.04 for remaining whole-genome sequences) with default settings.

### Genome analysis.

Contigs were analyzed and plots were created using the CMG-biotools package as described in the work of Vesth et al. (39). Briefly, genes were predicted using Prodigal 2.00. Gene families were constructed by genome-wide and pairwise BLAST comparisons. Genes were considered part of the same gene family with a sequence identity of >50% over at least 50% of the length of the longest gene. A pan-genomic dendrogram based on occurrences of gene families was used to sort input order by clustering prior to generating the pan- and core-genome plots (14).

Putative biosynthetic pathways were predicted from sequences (FASTA) with antiSMASH 2.0 (8, 9), with KS and C domains of PKS and NRPS predicted with NaPDoS (10) using default settings. Pathways were assessed as being similar OBUs when MultiGeneBlast (81) analyses revealed that 80% of the genes in the pathway were present with homologues that show at least 60% amino acid identity. For assessment and assembly of pathways split between different contigs, the sequences of homologues on the same contig were used as the scaffold. MultiGeneBlast (81) was used for recursive OBU analysis across all 13 strains, thus providing pseudoscaffolds for larger pathways, which in turn give higher confidence in the assignments. Partial pathways with the same pattern of conservation were combined in order to avoid overestimation of diversity. Predicted genes involved in the putative indolmycin biosynthetic pathway are labeled *unk* for “unknown.”

### Mapping of genes shared by groups of strains.

All predicted sets of protein sequences for the 13 strains were compared using the blastp function from the BLAST+ suite (82). These 169 whole-genome BLAST tables were analyzed to identify bidirectional best hits in all pairwise comparisons. Using custom Python scripts, this output was analyzed to identify, for all proteins, the strains in which orthologs were found. This allowed identification of unique genes, genes shared by clades and subclades of species, and genes shared by all 13 strains of *Pseudoalteromonas*. The script also generates a binary 13-digit barcode of the presence/absence of gene orthologs across the 13 strains for all proteins in the pan-genome.

### Nucleotide sequence accession numbers.

 The whole-genome shotgun projects have been deposited at GenBank under the accession numbers AUXS00000000, AUXT00000000, AUXU00000000, AUXV00000000, AUXX00000000, AUXY00000000, AUXZ00000000, AUYA00000000, AUYB00000000, and AUYC00000000. The versions described in this paper are versions AUXS01000000, AUXT01000000, AUXU01000000, AUXV01000000, AUXX01000000, AUXY01000000, AUXZ01000000, AUYA01000000, AUYB01000000, and AUYC01000000.
