# Supplementary material for: An Integrated Metabolomic and Genomic Mining Workflow To Uncover the Biosynthetic Potential of Bacteria
Source: mSystems. 2016 May 3;1(3):e00028-15. doi: 10.1128/mSystems.00028-15 (PMC5069768; doi:10.1128/mSystems.00028-15)
Supplement: Figure S1 [file sys003162020sf2.docx]

**Supplementary Information for An Integrated Metabolomic and Genomic Mining Workflow to Uncover the Biosynthetic Potential of Bacteria**

**Figure S1. Filtered pan- and core-metabolome plots**

***
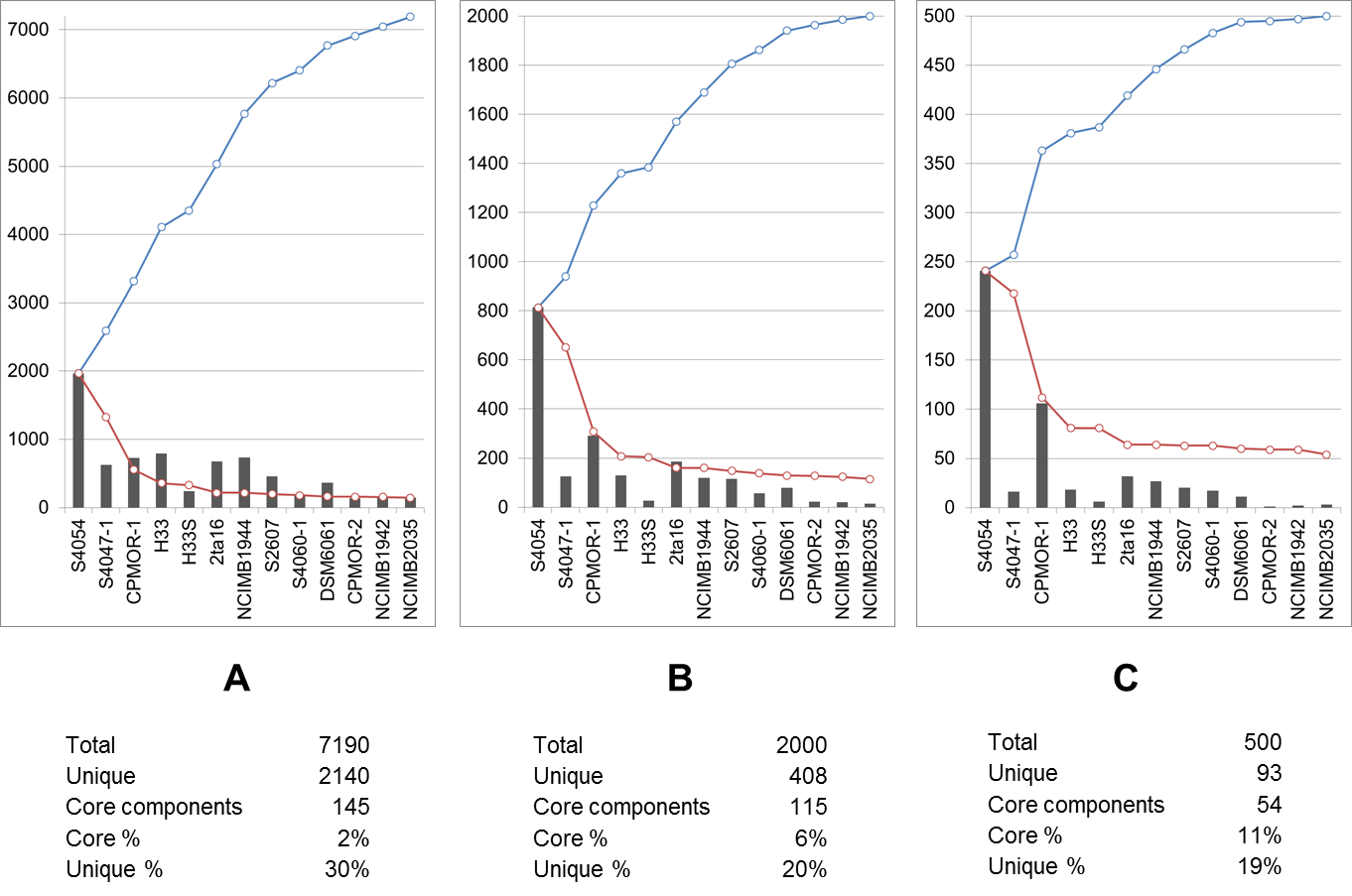
***

**Fig. S1.** A) The pan-metabolome curve (blue) connects the cumulative number of the total number of molecular features detected (positive and negative mode merged). The core-genome curve (red) connects the conserved number of features. The bars show the number of new molecular features detected in each extract (media components excluded). B) The pan- (blue) and core-(red) metabolome curves of the 2,000 most intense features. C) The pan- (blue) and core-(red) metabolome curves of the 500 most intense features.
