# Supplementary material for: An Integrated Metabolomic and Genomic Mining Workflow To Uncover the Biosynthetic Potential of Bacteria
Source: mSystems. 2016 May 3;1(3):e00028-15. doi: 10.1128/mSystems.00028-15 (PMC5069768; doi:10.1128/mSystems.00028-15)
Supplement: Table S1 [file sys003162020st7.docx]

**Supplementary Information for An Integrated Metabolomic and Genomic Mining Workflow to Uncover the Biosynthetic Potential of Bacteria**

**Table S1. Overall genomic features of the 13 *P. luteoviolacea* strains**

**Table S1.** Overall descriptive features of all 13 draft genomes. Total genes predicted using Prodigal 2.00, while antiSMASH 2.0 (Medema et al. 2011, Blin et al. 2013) was used to predict the number of genes allocated to secondary metabolism. *The total number of OBUs (in parentheses) and number of PKS/NRPS pathways were calculated based on antiSMASH and NaPDoS (Ziemert et al. 2012) predictions and recursive analysis by MultiGeneBlast (Medema et al. 2013).

Medema MH, Blin K, Cimermanicic P, de Jager V, Zakrzewski P, Fischbach MA, Weber T, Takano E and Breitling R (2011) antiSMASH: rapid identification, annotation and analysis of secondary metabolite biosynthesis gene clusters in bacterial and fungal genome sequences. *Nucleic Acids Res* 39:W339–46.

Blin K, Medema MH, Sazempour D, Fischbach MA, Breitling R, Takano E and Weber T (2013) antiSMASH 2.0--a versatile platform for genome mining of secondary metabolite producers. *Nucleic Acids Res* 41:W204–12.

Ziemert N, Podell S, Penn K, Badger JH, Allen E and Jensen PR (2012) The natural product domain seeker NaPDoS: a phylogeny based bioinformatic tool to classify secondary metabolite gene diversity. *PLoS One* 7:e34064. A

Medema MH, Takano E, Breitling R (2013) Detecting sequence homology at the gene cluster level with MultiGeneBlast. *Mol Biol Evol* 30:1218–23.
