# Supplementary material for: An Integrated Metabolomic and Genomic Mining Workflow To Uncover the Biosynthetic Potential of Bacteria
Source: mSystems. 2016 May 3;1(3):e00028-15. doi: 10.1128/mSystems.00028-15 (PMC5069768; doi:10.1128/mSystems.00028-15)
Supplement: Table S2 [file sys003162020st8.docx]

**Supplementary Information for An Integrated Metabolomic and Genomic Mining Workflow to Uncover the Biosynthetic Potential of Bacteria**

**Table S2. Overview of predicted Operational Biosynthetic Units (OBUs)**

**Table S2.** Pathway (OBU) distributions among the 13 *Pseudoalteromonas luteoviolacea* strains and their tentative functionality as predicted by antiSMASH. * Marks partial pathways on split contigs. Partial pathways with the same pattern of conservation are combined in order to avoid overestimation of diversity.
