# Supplementary material for: An Integrated Metabolomic and Genomic Mining Workflow To Uncover the Biosynthetic Potential of Bacteria
Source: mSystems. 2016 May 3;1(3):e00028-15. doi: 10.1128/mSystems.00028-15 (PMC5069768; doi:10.1128/mSystems.00028-15)
Supplement: Figure S2 [file sys003162020sf3.docx]

**Supplementary Information for An Integrated Metabolomic and Genomic Mining Workflow to Uncover the Biosynthetic Potential of Bacteria**

**Figure S2a. Full molecular network**


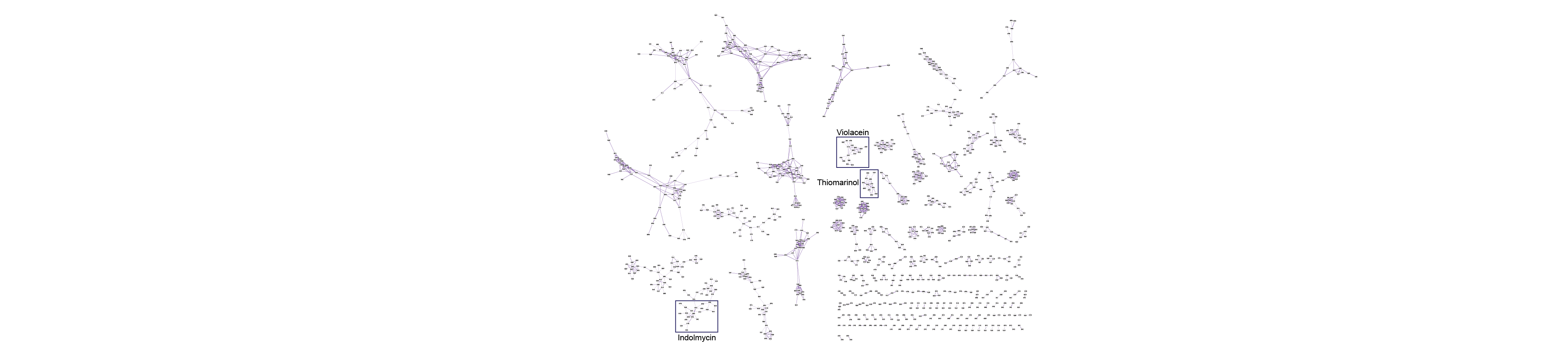


**Fig. S2a.** Molecular network of 13 strains of *P. luteoviolacea* based on LC-ESI^+^-MS/MS. Spectra originating from blank media samples are excluded from the analysis. Highlighted are the three gene cluster family-molecular family pairs identified in this study, those are violacein, indolmycin, and thiomarinol.

**Figure S2b. Network of the violacein molecular family**


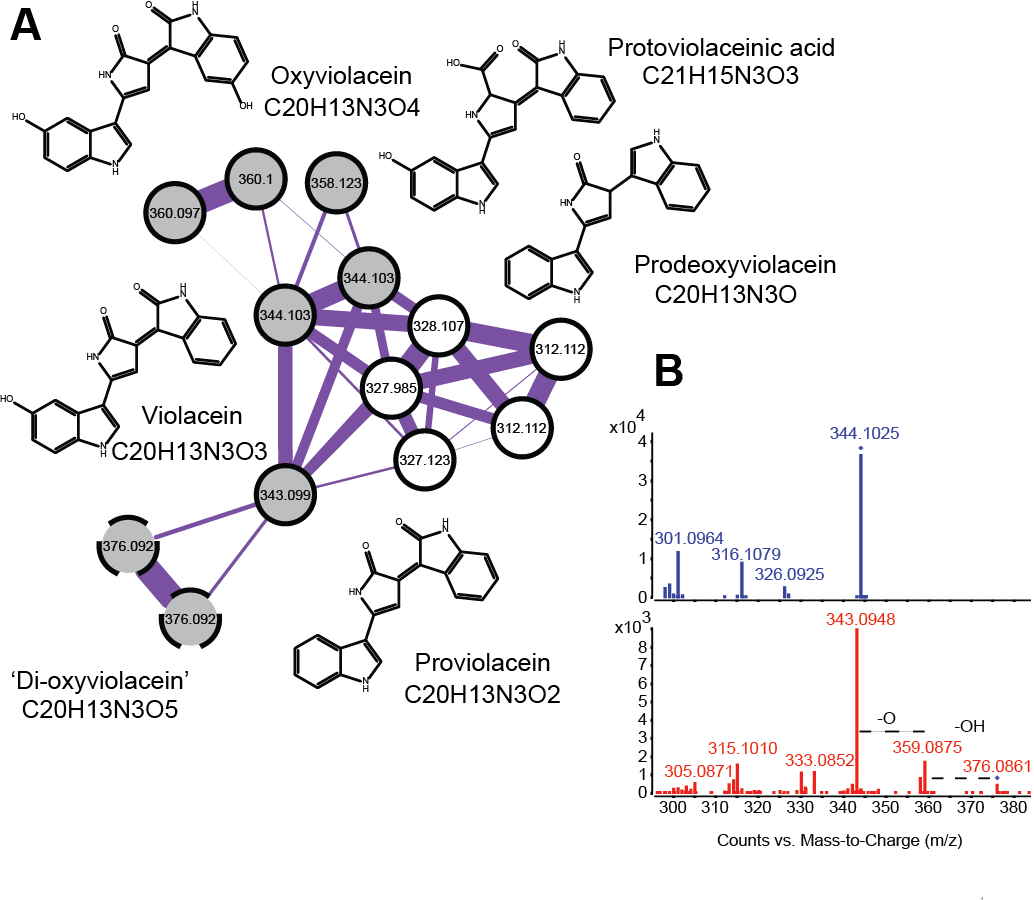


**Fig. S2b.** A) Molecular network of the violacein MF. Grey nodes are shared between all strains, while white nodes are shared but multiple, but not all strains. Dashed nodes indicate a novel analogue. B) Selected zoom of MS/MS spectra of violacein (top) with parent mass [M+H]^+^ 344 Da and the novel analogue (bottom) with an extra hydroxyl group [M+H]^+^ 376 Da.
