# Supplementary material for: An Integrated Metabolomic and Genomic Mining Workflow To Uncover the Biosynthetic Potential of Bacteria
Source: mSystems. 2016 May 3;1(3):e00028-15. doi: 10.1128/mSystems.00028-15 (PMC5069768; doi:10.1128/mSystems.00028-15)
Supplement: Table S3 [file sys003162020st9.docx]

**Supplementary Information for An Integrated Metabolomic and Genomic Mining Workflow to Uncover the Biosynthetic Potential of Bacteria Table S3. 50 discriminating molecular features identified by GA/SVM**

**Table S3 continued. 50 discriminating molecular features identified by GA/SVM**

**Table S3.** The 50 descriminating molecular features identified with GA/SVM from the 500 most intense features. Molecular formulas are determined with MassHunter function ‘Generate formulas’, also considering the isotope pattern of the peak. All tentative IDs are based on hits in AntiMarin or Metlin, and the candidates are evaluated based on accurate mass, isotope pattern (in particular for the halogenated compounds), relative retention time, and fragmentation pattern (for Metlin hits). Potential noise refers to unique masses that are detected by the software at high ion counts, yet it is not possible to confirm the identity of the molecular ion (and thus corresponding formulae and mass accuracy) by the presence of adducts or dimers.
