# Supplementary material for: An Integrated Metabolomic and Genomic Mining Workflow To Uncover the Biosynthetic Potential of Bacteria
Source: mSystems. 2016 May 3;1(3):e00028-15. doi: 10.1128/mSystems.00028-15 (PMC5069768; doi:10.1128/mSystems.00028-15)
Supplement: Table S4 [file sys003162020st10.docx]

**Supplementary Information for An Integrated Metabolomic and Genomic Mining Workflow to Uncover the Biosynthetic Potential of Bacteria Table S4. Halogenated molecular features found by mass defect screening**

**Table S4 continued. Halogenated molecular features found by mass defect screening**

|  |
| --- |

**Table S4.** List of halogenated molecular features identified by mass defect screening in MassHunter. The expected mass defect (0.0937 Da with -0.02 Da per 100 Da +/- 0.0100 Da) was determined from known halogenated compounds from *Pseudoalteromonas* in AntiMarin. The isotope pattern was used to confirm the presence of halogenations and used to calculate the molecular formula. Tentative IDs are based on hits in AntiMarin and evaluated based on accurate mass and isotope pattern. Compound marked * have no hit but belong to a known class of isomeric compounds. ** Peaks have a poor isotope match resulting in ambiguous determination of the formula.
