# Supplementary material for: An Integrated Metabolomic and Genomic Mining Workflow To Uncover the Biosynthetic Potential of Bacteria
Source: mSystems. 2016 May 3;1(3):e00028-15. doi: 10.1128/mSystems.00028-15 (PMC5069768; doi:10.1128/mSystems.00028-15)
Supplement: Figure S3 [file sys003162020sf4.docx]

**Supplementary Information for An Integrated Metabolomic and Genomic Mining Workflow to Uncover the Biosynthetic Potential of Bacteria**

**Figure S3a. Synteny map of *bmp* pathway**

**
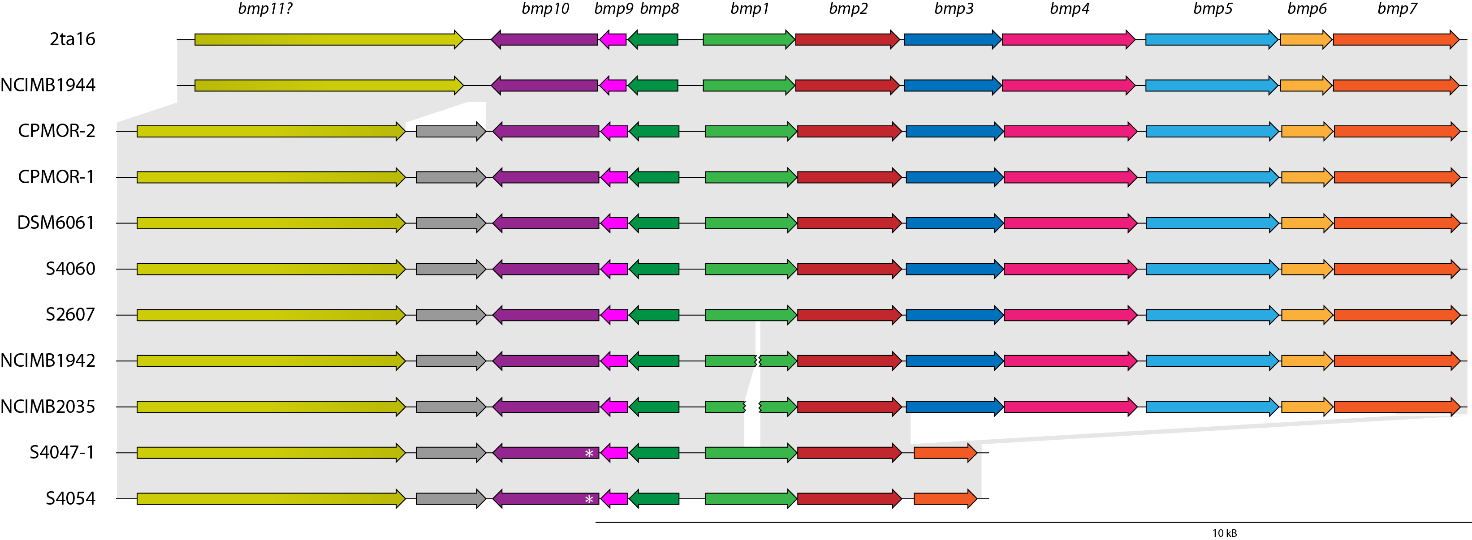
**

**Fig 3a*.*** Synteny maps of the *bmp*-cluster in the genomes of 11 isolates. In two isolates, H33 and H33S, no trace of the pathway was found. Note that in NCIMB1942 and NCIMB2035, the cluster was found distributed across two contigs, in both cases with the gap being in the *bmp1* gene. While this could be a sequencing artifact, no halogenated compounds were found in these two isolates. For S4047-1 and S4054, *bmp3-6* and a part of *bmp7* was missing, as well as point mutations (or sequencing errors in *bmp10*. In all strains, a predicted multidrug transporter was found downstream of *bmp10*, suggesting a possible resistance mechanism (tentatively names *bmp11*).

**Figure S3b. Synteny plot of *unk* gene cluster and indolmycin biosynthetic gene cluster**

**
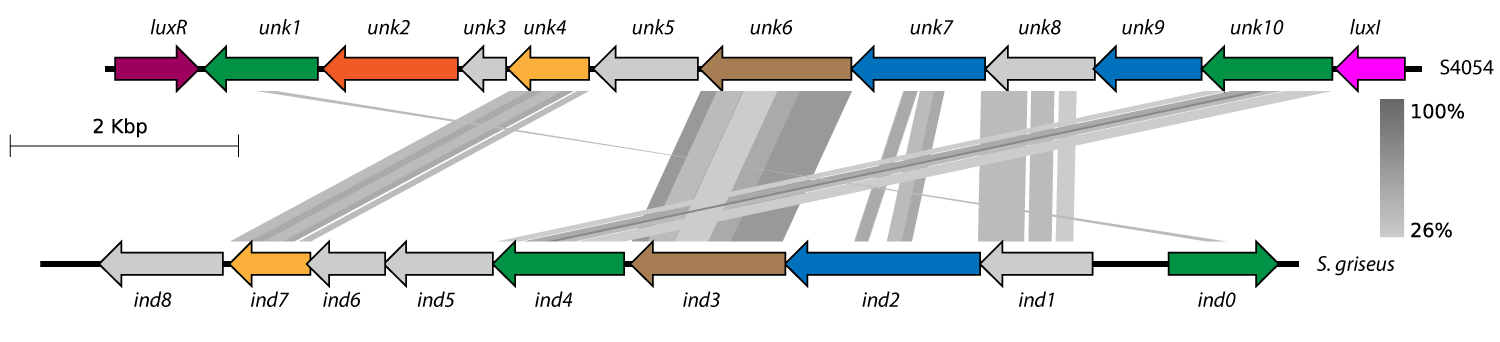
**

**Figure S3b.** Synteny plot of unk gene cluster from S4044 and indolmycin biosynthetic gene cluster from Streptomyces griseus subsp. griseus strain ATCC 12648. The figure was constructed using Easyfig 2.2.2 and the tblastx option (Sullivan, Petty, & Beatson, 2011). The BLAST options were set to minimum length 25, minimum identity to 25% and max E value to 0.001.

Sullivan MJ, Petty NK, and Beatson SA (2011). Easyfig: a genome comparison visualizer. *Bioinformatics*, *27*(7), 1009–1010. http://doi.org/10.1093/bioinformatics/btr039
