# Supplementary material for: An Integrated Metabolomic and Genomic Mining Workflow To Uncover the Biosynthetic Potential of Bacteria
Source: mSystems. 2016 May 3;1(3):e00028-15. doi: 10.1128/mSystems.00028-15 (PMC5069768; doi:10.1128/mSystems.00028-15)
Supplement: Figure S4 [file sys003162020sf5.docx]

**Supplementary Information for An Integrated Metabolomic and Genomic Mining Workflow to Uncover the Biosynthetic Potential of Bacteria**

**Figure S4. Tentative identification of dimeric halogenated compounds**

***
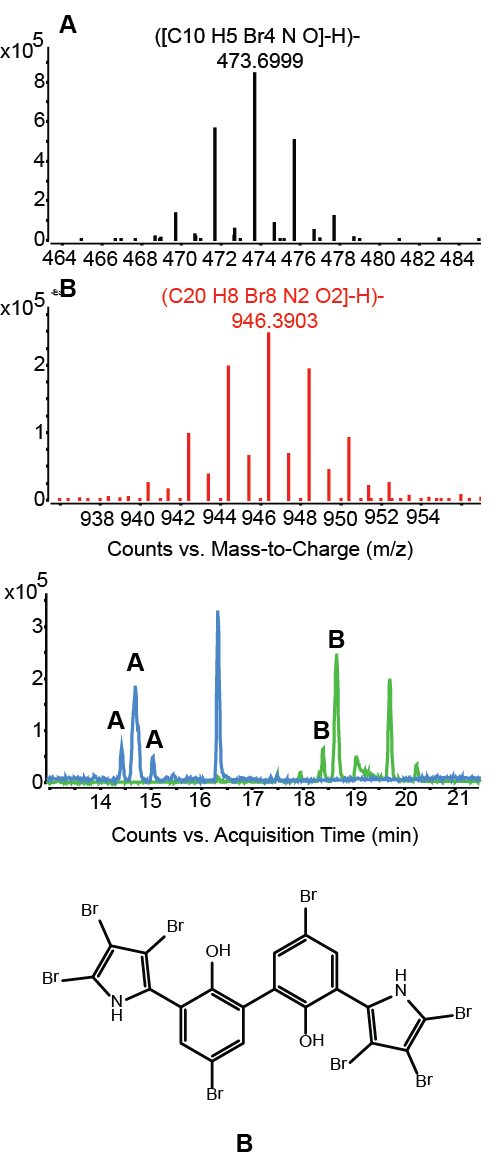
***

**Fig. S4.** Isotope patterns of A) C_10_H_5_Br_4_NO (RT 14.42, 14.63, and 14.99 min) and B) C_20_H_8_Br_8_N_2_O_2_ (RT 18.39 + 18.66 min) detected in ESI^-^ (top) and the corresponding EIC (bottom) and putative structure of a ‘bis-tetrabromopseudilin’.
