# Supplementary material for: An Integrated Metabolomic and Genomic Mining Workflow To Uncover the Biosynthetic Potential of Bacteria
Source: mSystems. 2016 May 3;1(3):e00028-15. doi: 10.1128/mSystems.00028-15 (PMC5069768; doi:10.1128/mSystems.00028-15)
Supplement: Figure S5 [file sys003162020sf6.docx]

**Supplementary Information for An Integrated Metabolomic and Genomic Mining Workflow to Uncover the Biosynthetic Potential of Bacteria**

**Figure S5. Network of the indolmycin molecular family**
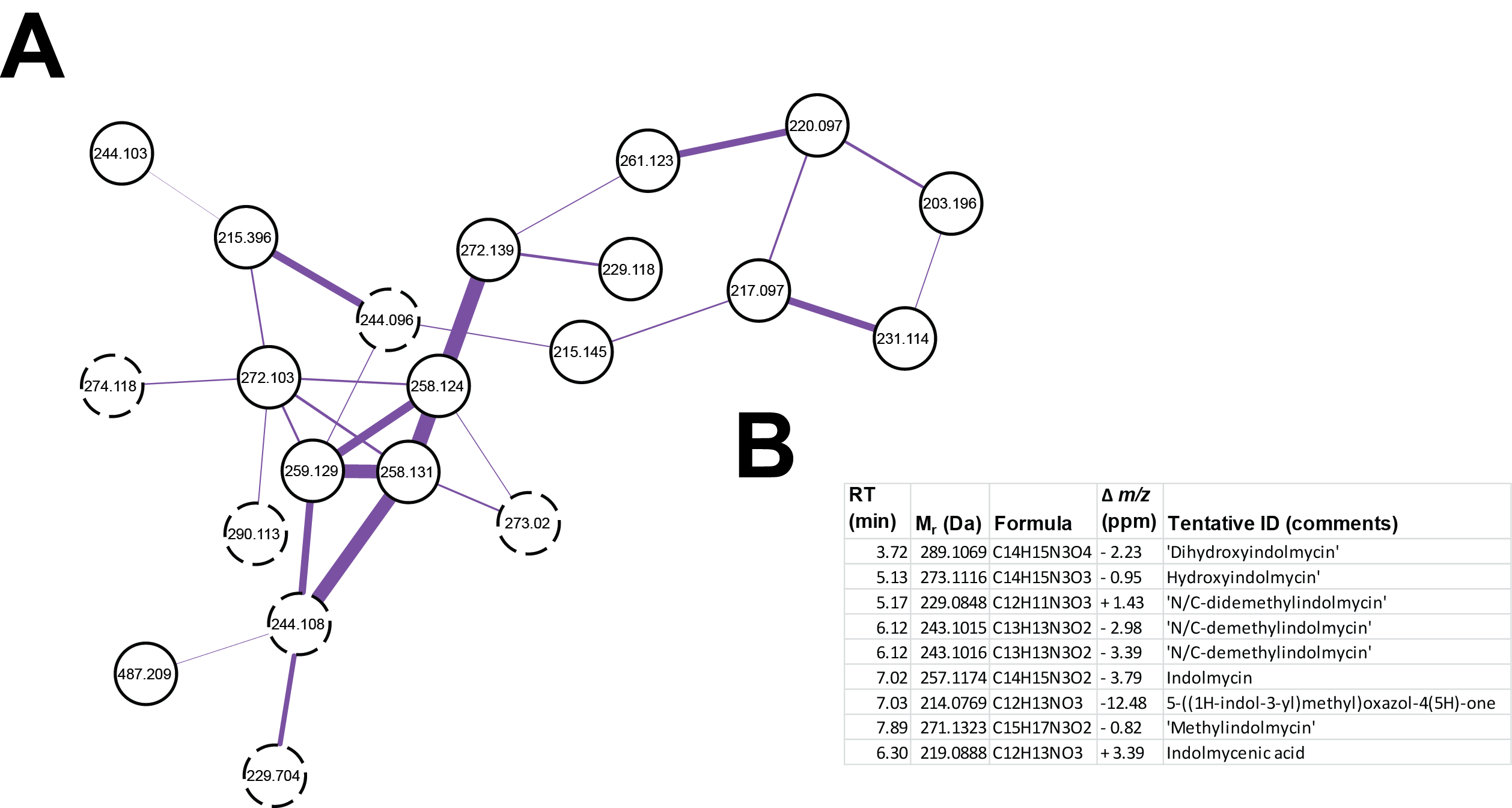


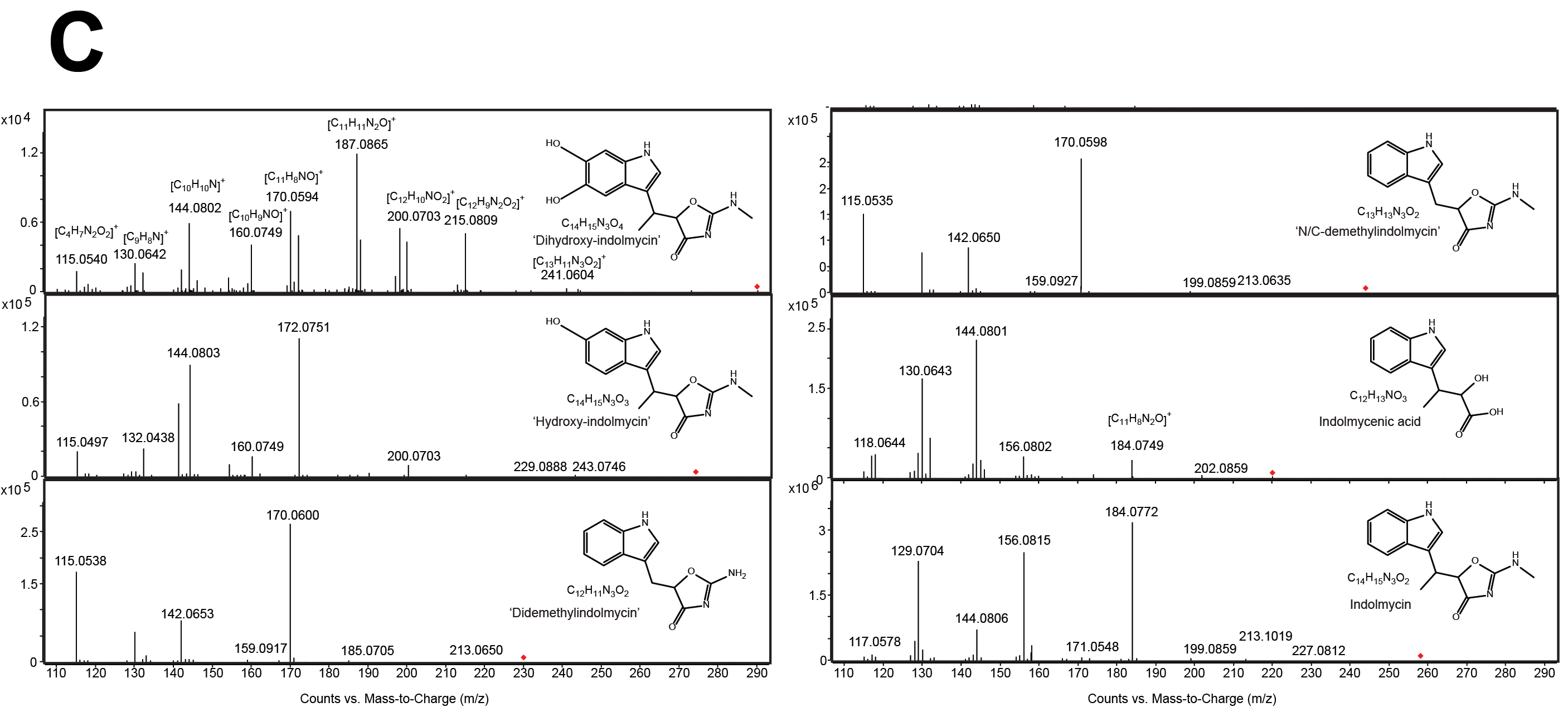


**Fig. S5*.*** A) Molecular network of the indolmycin molecular family. Dashed nodes indicate a novel analogue. B) Tentatively identified indolmycin analogues in strains S4047-1, S4054, and CPMOR-1. C) MS/MS spectra of selected analogues with assigned fragments.
