# Supplementary material for: An Integrated Metabolomic and Genomic Mining Workflow To Uncover the Biosynthetic Potential of Bacteria
Source: mSystems. 2016 May 3;1(3):e00028-15. doi: 10.1128/mSystems.00028-15 (PMC5069768; doi:10.1128/mSystems.00028-15)
Supplement: Text S1 [file sys003162020s1.docx]

**Supplementary Information for An Integrated Metabolomic and Genomic Mining Workflow to Uncover the Biosynthetic Potential of Bacteria**

**Supplementary Materials and Methods:**

**Strain cultivation and extraction.** The strains were cultured in biological duplicates in 20 mL Marine Broth at 25 ºC (200 rpm) for 48h. Cultures were extracted with 20 mL ethyl acetate (EtOAc) with 0.1% formic acid (FA), ultrasonicated for 10 min, and left on a shaking table (100 rpm) for 30 min. Phases were separated by centrifugation (3000 rcf, 4 ºC, 15 min). The cultures were re-extracted with 10 mL butanol (BuOH). The supernatants were pooled, dried under nitrogen, and re-dissolved in 2 mL methanol (MeOH). Samples for LC-MS/MS and molecular networking were used directly (1 µL injection), while samples for LC-MS and untargeted feature extraction were diluted 20-fold before injection (3 µL injection).

**LC-MS and LC-MS/MS data acquisition.** LC-MS and MS/MS analyses were performed on an Agilent 6550 iFunnel Q-TOF LC-MS (Agilent Technologies, Santa Clara, CA, US) coupled to an Agilent 1290 Infinity UHPLC system equipped with a Flexible Cube module. Compounds were separated on a Poroshell 120 phenyl-hexyl column (Agilent, 250 mm × 2.1 mm, 2.7 µm) at 60 °C with a water-acetonitrile (AcCN) gradient (both buffered with 20 mM formic acid (FA)) running from 10-100% AcCN over 20 min followed by a 4 min wash (100% AcCN). The gradient was then returned to 10% AcCN for a total gradient time of 26 min. Data was recorded both in positive and negative electrospray (ESI) mode and data was acquired in the *m/z* 100-1,700 Da mass range with a sampling rate of 2 Hz. The instrument was tuned and calibrated using a proprietary Agilent calibration algorithm using the Agilent ESI-L tuning mix solution. During operation, a lock mass solution containing ions *m/z* 119.9881 and 966.0007 in negative and *m/z* 186.2216 and 922.0098 in positive was constantly infused.

Data for molecular networking was collected using a data-dependent ESI^+^-LC-MS/MS as reported previously (Kildgaard et al. 2014) with the following modifications. MS1 spectra were recorded in positive electrospray mode from *m/z* 200-1,700 Da followed by MS/MS with a fixed collision energy of 25 V and a speed of 5 scans/sec. Spectra were obtained for the three most intense ions, which were excluded after being detected twice; however, released after 0.5 min for detection of analogues with different retention times.

Due to carry-over in the auto sampler of certain compounds (polybrominated), the samples were split in to two groups to minimize carry-over, i.e. non- and positive PBP producers (Vynne et al. 2012). Within the two groups, the samples were randomized using the macro developed by Bertrand *et al.* (2013) with blank runs every 5 samples and blank media control samples every 10 samples to assess the extent of the carry-over throughout the batch. Extensive valve cleaning was applied during the run. Likewise, the Flexible Cube solvents were 20% dichloromethane in 2-propanol (v/v%) and 30% water in 2-propanol to maximize removal of problematic compounds.

Kildgaard S, Mansson M, Dosen I, Klitgaard A, Frisvad JC, Larsen TO and Nielsen KF (2014) Accurate dereplication of bioactive secondary metabolites from marine-derived fungi by UHPLC-DAD-QTOFMS and a MS/HRMS library. *Mar Drugs* 12:3681–705.

Vynne NG, Mansson M, Gram L (2012) Gene sequence based clustering assists in dereplication of Pseudoalteromonas luteoviolacea strains with identical inhibitory activity and antibiotic production. *Mar Drugs* 10:1729–40.

Bertrand S, Schumpp P, Bohni N, Bujard A, Azzollini A, Monod M, Gindro K and Wolfender JL (2013) Detection of metabolite induction in fungal co-cultures on solid media by high-throughput differential ultra-high pressure liquid chromatography-time-of-flight mass spectrometry fingerprinting. *J Chromatogr A* 1292:219–28.

**Feature extraction and multivariate analysis.** To deconvolute the raw total ion current spectra, the data-analysis program MassHunter (Agilent Technologies, v. B06.00) was used. Chemical features were extracted from the LC-MS data using the Molecular Features Extraction (MFE) algorithm and the recursive analysis workflow. Features were extracted from RT 2.00-21.00 min, with a minimum intensity of 5,000 counts and aligned considering adducts ([M+H]^+^, [M+Na]^+^, [M-H]^-^, [M+Cl/Br]^-^, [M+CH_3_COO]^-^) and neutral losses ([M-H_2_O]^+^). The isotopes of the chemical features were detected using a tolerance of 0.0025 *m/z* + 7 ppm error, and were limited to a charge state of 1, while compounds with an interminable charge were excluded. Feature alignment, binning, and alignment was performed using the following tolerances (Δ*m/z* 0.0025 ± 7 ppm), mass window set (±0.2 min, 15 ppm), and a MFE quality score of minimum 98. Only features present in both replicate samples were considered. For the recursive feature extraction, chromatograms were smoothed using a Gaussian function (3 point function width and 1.5 point Gaussian width) and a cut-off intensity of 3,500 counts was used. The threshold used for the MFE and recursive analysis was purposely set low to allow for the detection of numerous features to ensure correct alignment of peaks, after which the aligned feature list could be filtered based on a higher threshold.

Feature lists were imported to Genespring – Mass Profiler Professional (MPP) (Agilent Technologies, v. 12.6), and filtered for features with raw intensities lower than 100,000 (ESI^+^ data) and 60,000 counts (ESI^-^ data). Media components or other interfering signals were defined as peaks present in the medium blank and these were manually excluded from the analysis. Features present in all samples (including the blank), but having more than a 10x fold change between sample and medium blank were treated as potential carry-over and included on the ‘true compound’ feature list. The lists from ESI^+^ and ESI^-^ were merged in an Excel table as generic data and reimported into MPP, where features within RT ±0.15 min and 15 ppm mass tolerance were aligned. Intensities were normalized (quantile) and Z-transformed due to differences in intensities in ESI^+^ and ESI^-^. A total number of 8,699 features were aligned. By only taken in to account features present in both replicates, the number of features was reduced to 7,190. The list of discriminating features was generated in MPP using genetic algorithm with a population size of 25, 10 generations, and a mutation rate of 1. The GA was evaluated using the SVM with a linear kernel type with and imposed cost of 100 and ratio of 1. The feature list was validated via the leave-one-out method.

**Mass defect screening.** A list of all halogen containing compounds described from *Pseudoalteromonas* and *Alteromonas* was extracted from AntiMarin (v. 08.13). Based on this, the minimum mass defect from any metabolite was found to be 0.0937 Da, whilst the lowest mass defect increase per 100 Da was found to be 0.0263 Da. Chemical features were extracted using the same settings as for the MFE analysis, and then filtered for compounds with a mass defect of -0.0937 Da with -0.02 Da per 100 Da at a tolerance of +/- 0.0100 Da. Likewise, the listed was validated by the isotope patterns of the filtered features.
